# Systemic Administration of Curcumin Affect Anxiety-Related Behaviors in a Rat Model of Posttraumatic Stress Disorder via Activation of Serotonergic Systems

**DOI:** 10.1155/2018/9041309

**Published:** 2018-06-19

**Authors:** Bombi Lee, Hyejung Lee

**Affiliations:** Acupuncture and Meridian Science Research Center, College of Korean Medicine, Kyung Hee University, Seoul 02447, Republic of Korea

## Abstract

Posttraumatic stress disorder (PTSD) is a trauma-induced psychiatric disease characterized by impaired hyperarousal, fear extermination, depression, anxiety, and amnesic symptoms that may include the release of monoamines in the dread circuit. Curcumin (CUR), a major diarylheptanoid and polyphenolic component of* Curcuma longa*, reportedly possesses several pharmacological features, including antidiabetic, antiatherosclerotic, anticancer, and neuropsychiatric actions. But the anxiolytic-like effects of CUR and its mechanism of action in PTSD are unclear. The current research measured some anxiety-related behavioral responses to examine the effects of CUR on symptoms of anxiety in rats after single prolonged stress (SPS) exposure by reversing the serotonin (5-HT) dysfunction. Rats received CUR (20, 50, or 100 mg/kg, i.p., once daily) for 14 days after SPS exposure. Administration of CUR significantly increased the number of central zone crossings in the open field test and reduced grooming behavior in the elevated plus maze (EPM) test and increased the number of open-arm visits on the EPM test. CUR administration significantly reduced freezing response to contextual fear conditioning. CUR recovered neurochemical abnormalities and SPS-induced decreased 5-HT tissue levels in the hippocampus, amygdala, and striatum. These results suggested that CUR has anxiolytic-like effects on biochemical and behavioral symptoms associated with anxiety. Thus, CUR may be a useful agent to alleviate or treat psychiatric disorders similar to those observed in patients with PTSD.

## 1. Introduction

Posttraumatic stress disorder (PTSD) is a trauma-induced psychiatric disorder characterized by the obtrusive reexperiencing of past trauma, negative cognitions, avoidance, hyperarousal, and fear [1]. The characteristic signs of PTSD include fear, insensibility, hyperarousal, and nightmares, leading to an intense social responsibility due to high rates of comorbidity with anxiety and psychosocial impairment [2, 3].

Single prolonged stress (SPS) is a well-documented animal model of PTSD, as rats exposed to a SPS enhanced dysregulation of the hypothalamic-pituitary-adrenal (HPA) axis [4, 5], increased fear condition on the elevated plus maze (EPM) test, and increased anxiety-like responses [6]. These responses imitated the clinical symptoms observed in PTSD patients [7]. Thus, many researchers have shown that inadequate adaptation of the HPA axis can lead to pathological states of PTSD, producing anxiety-like symptoms [8].

Dysregulation of serotonin (5-HT) and production are impaired in patients with PTSD [9]. Fluoxetine (FLX) is an antidepressant known to be effective for treating patients with PTSD [10]. Several researchers indicated that FLX increases fear extinction and synaptic plasticity via a serotonergic pathway, which alters 5-HT level in the brain [11]. Although selective serotonin reuptake inhibitors, such as FLX, are efficacious in many patient, they have destructive effects that limit their use, including psychiatric disorders, such as loss of body weight, sexual dysfunction, and sedation [12]. Therefore, there is progressing need for encouraging treatments for PTSD [13]. Thus, many studies have been devoted to the use of new natural medicines that may be safer for long-term therapy [14].

Curcumin (CUR) is a major diarylheptanoid and polyphenol component of* Curcuma longa* with both medical and nutritional value, and CUR is extracted from the dry rhizome of* C. longa* Linn (Zingiberaceae), a perennial herb that is widely cultivated in tropical regions of Asia [15, 16]. CUR has been used for centuries in traditional medicine to treat a variety of inflammatory symptoms. In addition, CUR also significantly reverses stress-induced cognitive and behavioral changes in rats [15, 17, 18]. Some researchers have showed the profits of CUR on central nervous system functions including neuroprotective effects against depression-like behavior in animals [18–20]. In chronic menopause-induced ovariectomized animal, CUR protects against bone loss [21] and ameliorates spatial memory affected by aging in female animal [22]. Thus, CUR is a potential antistress with compound helpful effects on behavioral dysfunction [23], but its use as an alternative therapy to trauma-related disorders such as PTSD has been not evaluated, and it is unknown whether CUR can improve anxiety-like behaviors following SPS exposure in rats. The current study examined the medicinal effects of CUR on anxiety-like symptoms in rats exposed to SPS using the EPM test and the open field test (OFT) and the contextual fear conditioning and fear extinction, showing the symptoms of PTSD-related abnormalities. Moreover, we investigated how the behavioral effects were associated with the serotonergic system in the rats as an underlying mechanism.

## 2. Methods

### 2.1. Animals and Curcumin Administration

Seven-week-old adult male Sprague-Dawley rats (Samtako Animal Co., Seoul, Korea), weighing 220–245 g, were used in all of the experiments. The rats were housed in a limited access rodent facility with up to five rats per polycarbonate cage. The rats were sustained on a 12-h light/dark cycle (lights on at 7:00 a.m., lights off at 7:00 p.m.) under controlled temperature (23 ± 3°C) and relative humidity (54 ± 5 %) conditions. All of the animals adapted to this condition during the 7 days after their arrival. All of the methods were approved by the Animal Care and Use Committee of Kyung Hee University [KHUASP(SE)-15-115]. All of the procedures were executed according to the Guide for the Care and Use of Laboratory Animals issued by the Korea National Institute of Health.

**Figure 1:**
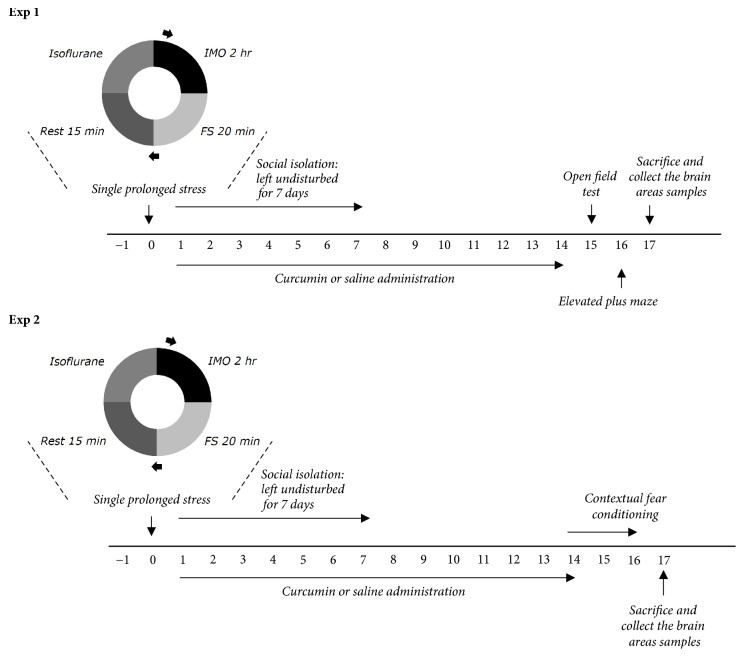
Experimental schedules for developing SPS-induced anxiety-like behaviors and curcumin treatment in the rats. Different groups of rats (n=6 or 7 animals per group) were used for all experiments.

After exposure to SPS, CUR (20, 50, and 100 mg/kg, body weight, Sigma-Aldrich Chemical Co. St. Louise, MO, USA) and fluoxetine (FLX, 10 mg/kg, fluoxetine hydrochloride; Sigma), which was used as the positive control, were intraperitoneally (i.p.) injected at a volume of 1 ml/kg for 14 days. The standard doses of CUR for rats considering the long-term treatment used in the present study was based on a previous study [15]. In total, six to seven animals were allotted to each group. CUR and FLX were dissolved in 0.9% saline before use. As a vehicle control, animals in the saline-treated (SAL) group were intraperitoneally given the equivalent volumes in saline without SPS exposure. Another group was pretreated with CUR (100 mg/kg, CUR group) without SPS exposure. The whole experimental schedules of SPS and behavioral experiments are shown in Figure 1.

### 2.2. Single Prolonged Stress

Animals were subjected to SPS according to a slightly modified version of the procedure described by Enman's groups [10, 24]. Briefly, rats were controlled for 120 min by a holder and then were subjected to a forced swimming condition for 20 min. The rats were dried and allowed to recuperate for 15 min and then were moved to a separate chamber where they were exposed to isoflurane until loss of consciousness (< 1 minute). Isoflurane exposure was conducted by placing a cotton ball soaked with isoflurane into an open conical tube and placed into the chamber (2–4 rats simultaneously). Rats were housed in pairs undisturbed for the next 7 days, with the exception of cage changes. All further experimental procedures began after the 7-day isolation period. Control animals were weighed daily and housed in pairs.

### 2.3. Measurement of Sucrose Intake

Intake of sucrose was measured according to a previously described method [10]. Briefly, a sucrose preference test, in which rats were housed in individual cage and given free access to two bottles containing 100 mL water and 100 mL sucrose solution (1%, w/v), was performed at 9:00 a.m. After 3 h, the volumes of consumed water and sucrose solution were measured, and the sucrose preference was calculated by the following formula: sucrose preference = sucrose consumption/( sucrose consumption + water consumption) × 100 %.

### 2.4. Elevated Plus Maze Test

The EPM test was carried out according to a method described previously [25]. Briefly, the rats were transferred to the EPM, which consisted of a 4-armed wooden platform in the form of a plus sign. The apparatus was raised 50 cm above the floor and was painted with black enamel. Two arms facing away from each other were protected, whereas the remaining two arms remained open. All arms were 50 cm in length, 10 cm in width, and joined in the center to create a 10 cm^2^ center platform. The video footage of these sessions was scored. At the start of each experiment, the rat could move freely for 5 min. The ratios of the total time spent in the close and open arms were used to measure anxiety.

### 2.5. Open Field Test

The rats completed the OFT before the EPM. The OFT was performed according to a previously described method [10, 25]. The open field area consisted of an enclosed square area made of dark opaque Plexiglas (60 × 60 cm) surrounded by walls (30 cm in height). All of the animal movements (locomotor activities) were determined with a computerized video-tracking system using the S-MART program (PanLab Co., Barcelona, Spain). The locomotor activity was measured in terms of total distance traveled in the container, and exploratory activity was measured based on evaluations of the total number of line crossings during 5 min. Painted white lines divided the area into 16 squares (15 × 15 cm each). Number of zone crossings and time spent in the central and peripheral zones were measured.

### 2.6. Contextual Fear Conditioning And Extinction

A separate group of rats that did not undergo the EPM testing (n = 4~5 for each group) was tested for contextual fear conditioning and extinction, after the SPS procedure. Contextual fear conditioning tests took place over 3 days and followed the procedures according to a method described previously [26]. Briefly, on the acquisition day, rats were placed in a foot shock chamber (26 cm wide × 30 cm long × 22 cm high) with an overhead camera and were allowed to explore freely for 120 s. After 120 s, a total of 10 electric shocks (0.75 mA, 2 s duration) were delivered at intervals of 74 s through the testing chamber floor. The rats then remained in the chamber for an additional 300 s with no shocks delivered. On test days 1-2, rats were placed for 8 min in the same chambers as on acquisition day, with no shocks delivered, to determine the extinction of contextual fear conditioning and extent of contextual fear learning. This behavior was chosen because it was previously shown to facilitate behavioral sensitization and to induce a reexperiencing of the aversive event. The percentage of freezing responses was calculated by dividing the freezing time by the total time. Freezing behavior was defined as complete immobility except for minor movements required for respiration.

### 2.7. Corticosterone, Tryptophan, and 5-HT Measurement

After the 15 days of rest designed to allow the development of PTSD, corticosterone (CORT) and tryptophan (TRP) in the plasma and 5-HT concentration in the brain tissue were assayed according to a previously described procedure [10, 25]. Four rats from experimental group were deeply anesthetized through inhalation with isoflurane (1.2 %) and were sacrificed one day after behavioral testing. The plasma was rapidly collected via the abdominal aorta, and the medial prefrontal cortex, amygdala, striatum, and hippocampus were quickly removed from the rat brain in a randomized order. Special care was taken to avoid presacrifice stress and the rats were killed quickly. CORT, TRP, and 5-HT levels were evaluated by a competitive enzyme-linked immunoassays (ELISAs) using a TRP antibody (Biocompare, San Francisco, CA, USA), a 5-HT antibody (Abcam, Cambridge, MA, USA), and a CORT antibody (Novus Biologicals, LLC, Littleton, CO, USA) according to the manufacturer's protocols.

### 2.8. Total RNA Isolation and Reverse Transcription-Polymerase Chain Reaction

The levels of tryptophan hydroxylase-1 (THP1) mRNA expression were measured by RT-PCR according to a previously described method [25]. In brief, total RNA was extracted from the hippocampus of each rat using RNAiso reagent (Life Technologies, Carlsbad, CA, USA) according to the manufacturer's instructions. cDNA was synthesized from 2 *μ*g total RNA using PrimeScript™ (Takara, Kyoto, Japan) with random hexamers (COSMO Genetech, Seoul, Korea). Then it was amplified at 58°C for 29 cycles to produce THP1 by PCR using TaKaRa Taq™ (Takara, Otsu, Japan) on a thermal cycler (MJ Research, Watertown, MA, USA). Data were normalized against glyceraldehyde 3-phosphate dehydrogenase (GADPH) expression in the corresponding sample.

### 2.9. Statistical Analysis

All of the measurements were performed by an independent investigator blind to the experimental conditions, and the results are expressed as mean ± standard error of the mean. Differences within or between normally distributed data were analyzed with analysis of variance (ANOVA) with SPSS (version 13.0; SPSS, Inc., Chicago, IL, USA) and Tukey's* post hoc *tests. Between-subjects two-way ANOVA was used to analyze the effects of CUR treatment and time. In all of the analyses, differences were considered statistically significant at* p value* < 0.05.

## 3. Results

### 3.1. Effects of Curcumin on Body Weight, Sucrose Intake, and Plasma Corticosterone Levels after Single Prolonged Stress Exposure

**Figure 2:**
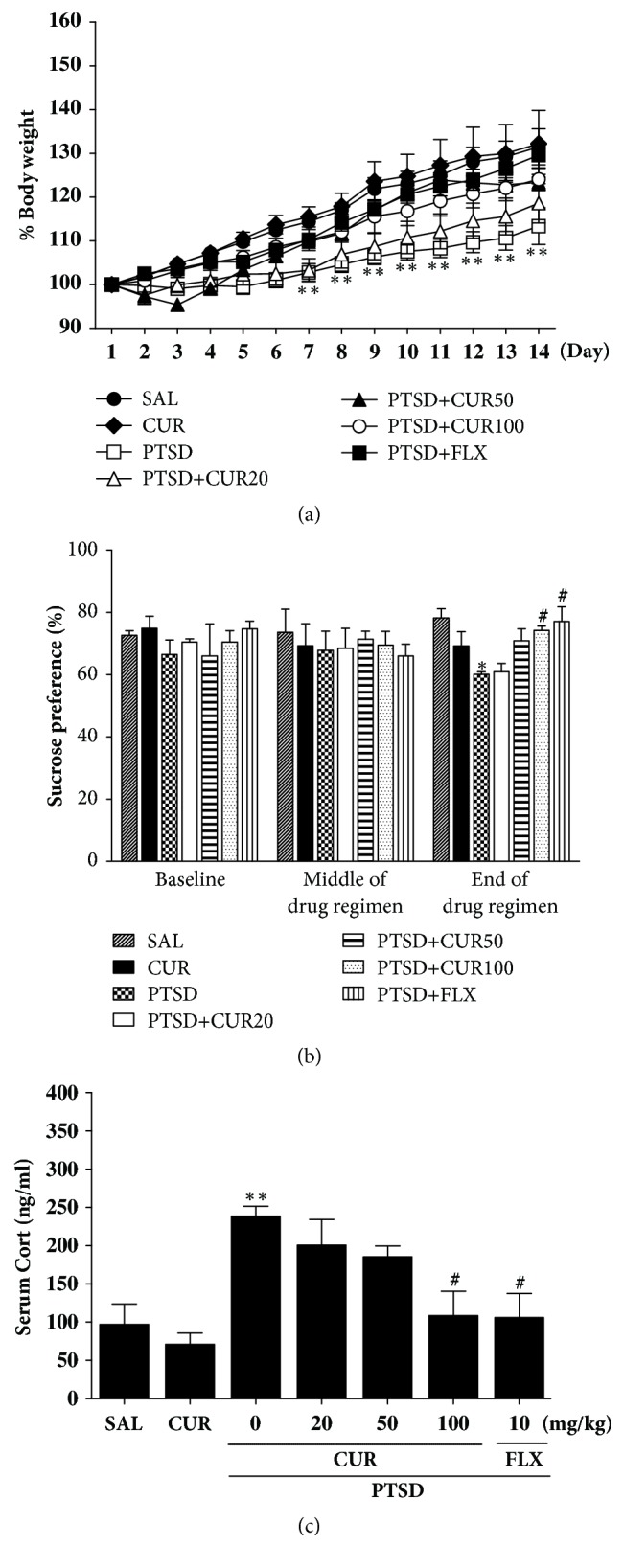
Effects of curcumin administration on body weights (a), sucrose intake (b), and plasma corticosterone levels (c) in rats exposed to SPS. ^*∗*^*p*<0.05 and ^*∗∗*^*p*<0.01* v*ersus SAL group; ^#^*p*<0.05* v*ersus PTSD group.

After SPS exposure, the body weight of each rat was measured for 14 days. Rats exposed to SPS began to lose body weight on the next day after SPS, and the rate of this stress-induced reduction in body weight was sustained or even, in some cases, increased, without a return to baseline (Figure 2(a)). The body weight of rats in the PTSD group reduced compared with that of the saline-treated (SAL) group (*p*<0.01). But 100 mg/kg of CUR inhibited loss of body weight compared with the PTSD group, although this result was only marginally statistically significant.

Anhedonia, a symptom of depression, was measured by the sucrose preference test 1 day before SPS (baseline), in the middle of the post-SPS period (7 days after SPS) and at the end of the drug regimen (14 day after SPS). Our analysis of sucrose intake revealed a significant gradual decrease in the rate at which sucrose intake decreased over the 14 days in the SPS compared to the saline-treated (SAL) group (*p*<0.05; Figure 2(b)). During this period, the sucrose intake of rats treated with 100 mg/kg of CUR did not show reduction as dramatically as did the sucrose intake of rats in the PTSD group (*p*<0.05). Also, the results also showed that recuperation in the sucrose intake of the PTSD+CUR100 group was almost comparable to that of the PTSD+FLX group.

ELISA analysis showed that rats that underwent SPS exposure had significantly increased plasma CORT concentration (245.87 %) compared to rats in the SAL group (*p*<0.01; Figure 2(c)). Therefore, the SPS process induced anxiety-like symptoms in rats, which was used to develop a PTSD model. However, treatment of CUR reduced the SPS-induced increase in plasma CORT concentrations (*p*<0.05).

### 3.2. Effects of Curcumin on Anxiety-Like Behaviors following Single Prolonged Stress Exposure

**Figure 3:**
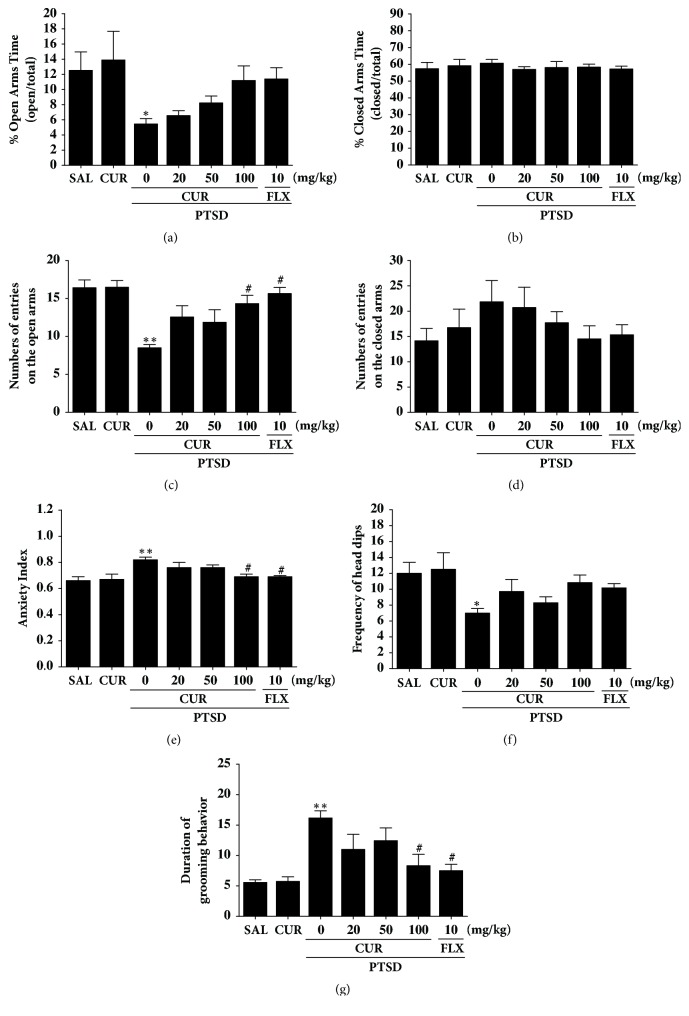
Effects of curcumin administration on the percentage of time spent in the open (a) and closed (b) arms, numbers of entries into open (c) and closed (d) arms, anxiety index (e), unprotected head dips (f), and grooming behavior (g) in the elevated plus maze test of rats exposed to SPS. ^*∗*^*p*<0.05 and ^*∗∗*^*p*<0.01* v*ersus SAL group; ^#^*p*<0.05* v*ersus PTSD group.

Rats displayed an apparent anxiety phenotype characterized by decreased open-arm exploration during the EPM test. Statistical analyses of the behavioral results showed that the percentage of time spent in the open arms of the maze significantly differed among the seven groups [F(6,42)=3.685,* p*<0.05](Figure 3(a)). The ANOVA also revealed a significant effect of the number of open-arm entries among the seven groups [F(6,42)=5.008,* p*<0.01] ](Figure 3(c)).* Post hoc* comparisons indicated that the number of entries and the percentage of time spent in the open arms of the maze decreased significantly in the PTSD group compared with the control group (*p*<0.05 and* p*<0.01). However, rats in the PTSD+CUR100 group showed significant restoration of the number of entries in the open arms of the maze compared with that of the PTSD group (*p*<0.05). The number of entries and the percentage of time spent in the closed arms of the maze did not differ significantly among the seven groups [F(6,42)=0.195,* p*=0.976, and F(6.42)=0.972,* p*=0.458](Figures 3(b) and 3(d)). CUR administration after SPS elicited anxiolytic and anxiogenic behavior. Our results indicated that increased number of entries in the open arms of the maze in the PTSD+CUR100 group was comparable to the exploratory behavior in the PTSD+FLX group. In total, the anxiety index, calculated based on the number of visits to and time spent in the open and closed arms, also differed among the seven groups of rats, with lower values in the CUR-treated rats (*p*<0.05; Figure 3(e)). Administering CUR significantly increased the frequency of defenceless head dips compared with that in the PTSD group, although this result was only marginally statistically significant (Figure 3(f)). However, the duration of grooming behavior was revered by 100 mg of CUR when administered after SPS exposure (*p*<0.05; Figure 3(g)).

### 3.3. Effects of Curcumin on Locomotion and Exploratory Behavior following Single Prolonged Stress Exposure

**Figure 4:**
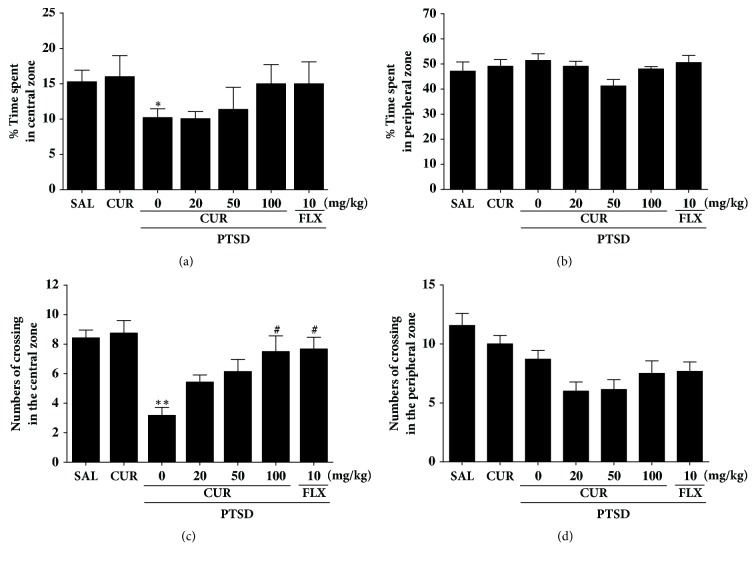
Effects of curcumin administration on locomotion and exploratory behavior in the open field test of rats exposed to SPS. Change in the time spent in central zone (a) and peripheral zone (b) and in the numbers of crossing in the central zone (c) and peripheral zone (d). ^*∗*^*p*<0.05 and ^*∗∗*^*p*<0.01* v*ersus SAL group; ^#^*p*<0.05* v*ersus PTSD group.

The OFT was used to assess the locomotion and exploratory behavior of the rats who underwent the SPS procedure (Figure 4). Rats exposed to SPS spent significantly less time in the central zone and correspondingly more time in the peripheral zone compared to the saline (SAL) group (*p*<0.05; Figure 4(a)). There was also a significant decrease in the number of central zone crossings following the SPS procedure (*p*<0.01; Figure 4(c)). Our results indicated that SPS-treated rats developed locomotion and exploratory activities that were closely associated with the anxiety-like behaviors observed in the OFT. However, the CUR-treated rats (100 mg/kg) displayed a significant increase in the number of central zone crossings compared with rats in the PTSD group (*p*<0.05), indicating that the exploratory-like behaviors of the PTSD+CUR100 group were similar to those of the PTSD+FLX group.

### 3.4. Effects of Curcumin on SPS-Induced Contextual Fear Conditioning and Extinction

**Figure 5:**
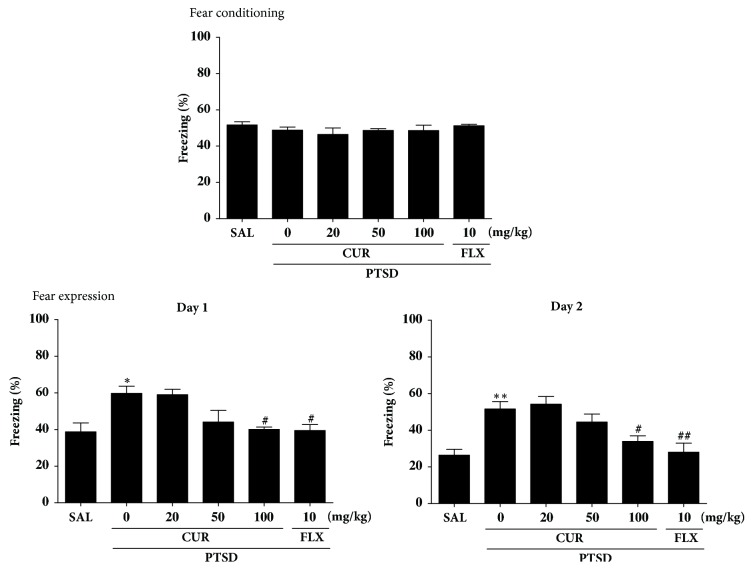
Effects of curcumin on freezing behavior from contextual fear conditioning testing after exposure to SPS in rats. The percentages of freezing time were determined on acquisition, day 1, and day 2. ^*∗∗*^*p*<0.01* v*ersus SAL group; ^#^*p*<0.05 and ^##^*p*<0.01* v*ersus SPS group.

The effects of CUR administration on the contextual freezing behavior of rats are shown in Figure 5. Unconditioned freezing duration in response to foot shock was not different between groups [F(5,27)=0.223,* p*=0.514]. In contrast, two-way ANOVA across the three testing sessions revealed a significant main effect of treatment [F(5,27)=10.13,* p*<0.05], a significant main effect of time [F(5,27)=28.11,* p*=0.714], and a significant interaction between treatment and time [F(5,27)=2.130,* p*<0.05] for freezing behavior. In the contextual freezing measurement, the freezing time significantly increased after exposure to SPS (*p*<0.05 on day 1 and* p*<0.01 on day 2). The percentage of time spent displaying freezing behavior was significantly decreased in the group treated with 100 mg/kg of CUR on days 1 and 2, respectively (*p*<0.05). These results indicate that a persistent fear response to the original context was associated with the traumatic events that occurred there and that repeated CUR treatment ameliorated the context-dependent freezing behavior in the rats.

### 3.5. Effects of Curcumin on the 5-HT Concentration in the Hippocampus following Single Prolonged Stress Exposure

**Figure 6:**
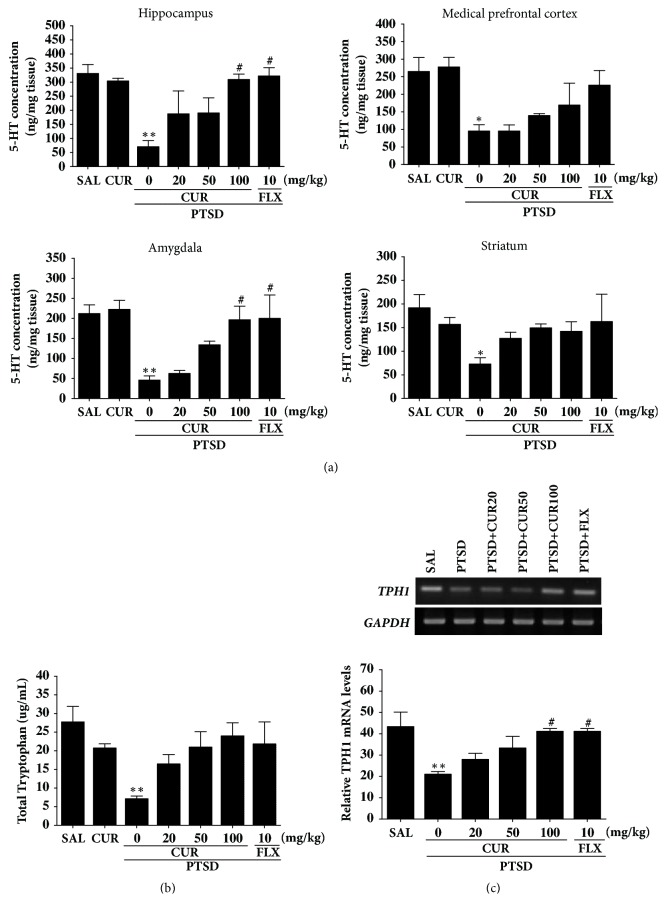
Effects of curcumin on the 5-HT concentration in the brain (a), plasma TRP levels (b), and expression of TPH1 mRNA (c) of rats exposed to SPS. PCR bands on agarose gels and relative intensities are shown. ^*∗*^*p*<0.05 and ^*∗∗*^*p*<0.01* v*ersus SAL group; ^#^*p*<0.05* v*ersus PTSD group.

Figure 6(a) shows differences in the regional concentration of 5-HT among the group. The* post hoc* test results indicated a significant decrease in 5-HT levels in the hippocampus of the PTSD group compared to the SAL group (*p*<0.01). Daily administration of CUR significantly inhibited the SPS-induced decrease in the 5-HT level in the hippocampus compared with the PTSD group (*p*<0.05). The* post hoc* test results indicated a significant decrease in 5-HT concentrations in the medial prefrontal cortex of the PTSD group compared to the SAL group (*p*<0.05). Daily administration of CUR significantly inhibited the SPS-induced decrease in the concentration of 5-HT in the medial prefrontal cortex compared with the PTSD group, although this result was only minimally significant. After treatment with CUR, the levels of 5-HT in the amygdala were significantly reversed, by 326.08 % of that in the PTSD group (*p*<0.05). Additionally, 5-HT concentrations in the brain regions of rats receiving 10 mg/kg of FLX were similar to those of rats receiving 100 mg/kg of CUR.

ELISA analysis showed that SPS exposure significantly decreased, by 25.69 %, the plasma TRP concentrations compared to rats in the SAL group (*p*<0.01; Figure 6(b)). However, administration of CUR inhibited the SPS-induced decrease in plasma TRP concentrations, although this result was only marginally statistically significant.

### 3.6. Effects of Curcumin on Expression of TPH1 mRNA in the Hippocampus following Single Prolonged Stress Exposure

To examine the effect of CUR on the level of TPH1 mRNA expression in rat hippocampus injured by SPS, the mRNA expression of TPH1 was analyzed by RT-PCR (Figure 6(c)). Although the mRNA level of TPH1 in the PTSD group significantly reduced compared with that in the SAL group (*p*<0.01), the expression levels in the PTSD group were similar to those in the SAL group after treatment with 100 mg/kg of CUR (*p*<0.05). This indicates that TPH1 mRNA levels in the hippocampus of rats receiving 100 mg/kg of CUR were similar to those of rats receiving 10 mg/kg of FLX.

## 4. Discussion

Administrating CUR after SPS exposure significantly decreased grooming behavior, reduced the anxiety index, and increased the number of open-arm visits on the EPM test. Administrating CUR after SPS also significantly decreased anxiety-like behaviors, as indicated by an increase in the number of central zone crossings in the central zone during the OFT. CUR administration significantly reduced freezing response to contextual fear conditioning. Thus, CUR appears to act as an anxiolytic. These results could lead to the development of novel therapeutics for the treatment of PTSD.

We investigated a range of CUR doses which appeared to have equivalent concentration of typical human consumption. Thus, we chose our highest dose as 100 mg/kg of body weight based on a previous study which showed that CUR exerted antidepressant effects at this dose in chronic stress-induced depression-like symptoms in rats [15]. According to the animal to human dose change formula using intestinal surface area, a rat injected with 100 mg/kg of body weight CUR is equivalent to an individual with 70 kg of weight strong 566 mg of CUR [27, 28]. While this computation is relatively crude, this dose is still significantly less than a typical commercially available CUR capsule which usually included approximately 1,000 mg of CUR. This is of significant importance as many previous clinical studies have used doses exceeding 2 g per day which required multiple daily intakes. While estimating animal data to human is difficult, it is important to retain the doses used in the animal study as close as possible to clinical situation.

A significant reduction in sucrose consumption was observed in SPS rats, consistent with previous studies [10]. There was also a significant positive correlation between sucrose intake and body weight (r=0.58) in the SPS rats. Thus, the reduced sucrose intake in SPS rats may reflect decreased response to rewards, anhedonia, suggesting that SPS procedures selected in the current results may produce anxiety-like symptoms. In this animal model, high CORT levels can develop anxiety by regulating anxiety-like behavior and enhancing dysregulation of the HPA axis [29], which may correlate with the progress of traumatic stress [29]. After SPS exposure, we observed a progressive reduction in body weight, an increase in plasma CORT concentration, and a reduction in sucrose preference compared to those before testing, indicating that SPS-induced anxiety-like symptoms were sufficiently stressful [30, 31]. Our research has shown that the beneficial effect of CUR depended largely on the period of drug administration, as the observed SPS-induced decrease in sucrose preference was reversed in the end but not the middle of the treatment in the sucrose preference test. Therefore, CUR had anxiolytic activity, suggesting that this treatment inhibits the HPA axis-associated behavioral and neurochemical responses caused by decreasing plasma CORT levels, which normalized psychological dysfunction.

Our behavioral investigation demonstrates the anxiolytic-like effects of CUR in an animal model. Administrating of CUR after SPS significantly reduced anxiety-like behaviors on the EPM test, as indicated by exploratory behaviors and more entries in the open arms [32]. Administrating CUR after SPS also significantly increases the number of central zone crossings in the OFT [25]. These changes showed to be specific and did not exert impaired locomotor activity because length of track in the EMP test and OFT was similar for all groups. Also, grooming behavior reveals that rats treated with CUR adapted better to novelty stress, consistent with previous results in the EMP test [29]. Also, CUR decreased the trauma stress-induced boosting potentiation of symptom to the novel stress of the EPM. Thus, because behaviors in the OFT and the EPM test are related to HPA axis-associated psychological symptom, our results indicated that CUR may inhibit HPA axis dysregulation.

Furthermore, consistent with previous studies, the results of this research indicate that the SPS model produces enhanced contextual freezing and anxiety-like behavior in the OFT, as well as stress-induced analgesia compared with that demonstrated by the control group [33]. The freezing behavior after SPS procedure also supported the notion that PTSD is characterized by an exaggerated reaction, which would be relevant for the original traumatic situation, to a mild stressor or to a reminder of the trauma [33]. Therefore, SPS exposure led to the acquisition of conditioned fear responses to trauma-related stimuli [34]. Our results support the idea that this PTSD model resembles the clinical symptoms of PTSD. Thus, the present results support that CUR could ameliorate fear memory and anxiety induced by traumatic stress.

Reexperiencing PTSD symptoms appears to lead to imbalance or dysfunctions of monoamines within the fear circuit areas of the medial prefrontal cortex, striatum, hippocampus, and amygdala [9]. We hypothesize that CUR modulates SPS-induced anxiety-like symptoms which are related to dysfunction of central 5-HT. In the present study, animals with PTSD treated with CUR had significant increase in 5-HT concentrations in the hippocampus and amygdala, and this may have inhibited the pathophysiology of PTSD. The effects of CUR can be reversed by manipulating 5-HT [9]. Thus, our results indicate that CUR, like FLX, restores the behavior and neurochemical alternations associated with anxiety by modulating serotonergic system in the brain [35].

Furthermore, we detected that SPS decreased the expression of TPH1 mRNA in the hippocampus of rat well as anxiety-like behaviors. CUR restored hippocampal TPH1 mRNA expression, suggesting that the modulation of serotonergic system plays a role in the anxiolytic effects of CUR.

There have been many clinical trials using CUR for various disorders and there has been no significant side effect reported. Because of its safety, many researchers suggested that CUR could be a good nontoxic alternative treatment instead of nonsteroidal anti-inflammatory drugs (NSAIDs). CUR has broad chemopreventive activity in preclinical studies and appears to be safe in animal and human studies [36]. For chemopreventive intervention studies to be successful, they must be provided in doses that are effective but be virtually free of toxicities. Some studies have suggested minimal toxicity with moderate doses of CUR given in various formulations. For example, two trials evaluating the efficacy of CUR for the treatment of arthritis or postoperative inflammation found that doses of 1,200 to 2,100 mg of CUR per day for 2–6 weeks were without adverse effects [37].

Some studies suggested that* Elettaria cardamomum* methanolic extract significantly recovered anxiety-like behaviors in a rat model of PTSD conducted by the OFT, EPM, and rotarod tests [38]. Also, administering berberine, an isoquinoline alkaloid derived from Korean traditional medicinal herbs, such as* Coptidis Rhizoma* after SPS procedure, significantly increased the time spent in the open arms in the EPM and prevented reduction in brain level of dopamine [39]. Therefore, herb-derived compounds could lead to the development of novel therapeutics for treating PTSD.

Interestingly, the PTSD+CUR100 group showed no significant recovery against the SPS-induced decrease in 5-HT levels in the medial prefrontal cortex and striatum, although the same dose of CUR produced statistically significant results in the activation of 5-HT level in the hippocampus and amygdala. Some studies demonstrated that, after SPS, there were area-dependent decreases of 5-HT in the medial prefrontal cortex, hippocampus, and amygdala, but not the striatum [9]. The fear circuit 5-HT is highly responsible for the PTSD-induced mood change and may explain why depression and anxiety symptoms occur frequently after traumatic events [40, 41]. In our results, only the 5-HT disturbances in the fear circuit areas can be reversed by CUR. The reason the high dose of CUR did not increase 5-HT level in the medial prefrontal cortex in the SPS-induced decrease 5-HT should be investigated further. Also, because the symptoms of PTSD occasionally appear long after the traumatic event, the schedule used in our experiments was unable to fully estimate the entire process of PTSD.

Many researchers have yet to achieve a satisfactory understanding of the etiology and neurobiological basis of PTSD. Many patients are resistant to improvement of their symptoms with treatment and never accomplish complete restoration. Thus, animal models of PTSD are of great value for understanding pathophysiology of PTSD and possibly in the development of novel, and potentially more effective, treatment or prevention strategies in PTSD through our study. In this study, we demonstrated the anxiolytic properties of CUR. We also observed the neuroprotective effects of CUR in a PTSD animal model. Based on these findings, we believe that CUR might be a potential candidate for anxiolytic or antidepressant therapy.

## 5. Conclusion

Administrating CUR after SPS exposure significantly increased the number of open-arm explorations on the EPM test, reduced the anxiety index, and decreased grooming behavior. Administrating CUR after SPS also significantly increased in the number of central zone crossings during the OFT. Therefore, administration of CUR was associated with anxiolytic-like effects in the EPM test and OFT, possibly by modifying serotonergic system. These findings indicated that CUR can ameliorate the neurochemical responses and psychological-related behaviors involved in anxiety. Thus, CUR may be an alternative treatment for preventing anxiety-like behavior associated with PTSD.
